# Supplementary material for: In-depth blood proteome profiling analysis revealed distinct functional characteristics of plasma proteins between severe and non-severe COVID-19 patients
Source: Sci Rep. 2020 Dec 29;10:22418. doi: 10.1038/s41598-020-80120-8 (PMC7772338; doi:10.1038/s41598-020-80120-8)
Supplement: Supplementary file 1 — Supplementary Figures. [file 41598_2020_80120_MOESM1_ESM.pptx]

## Slide 1
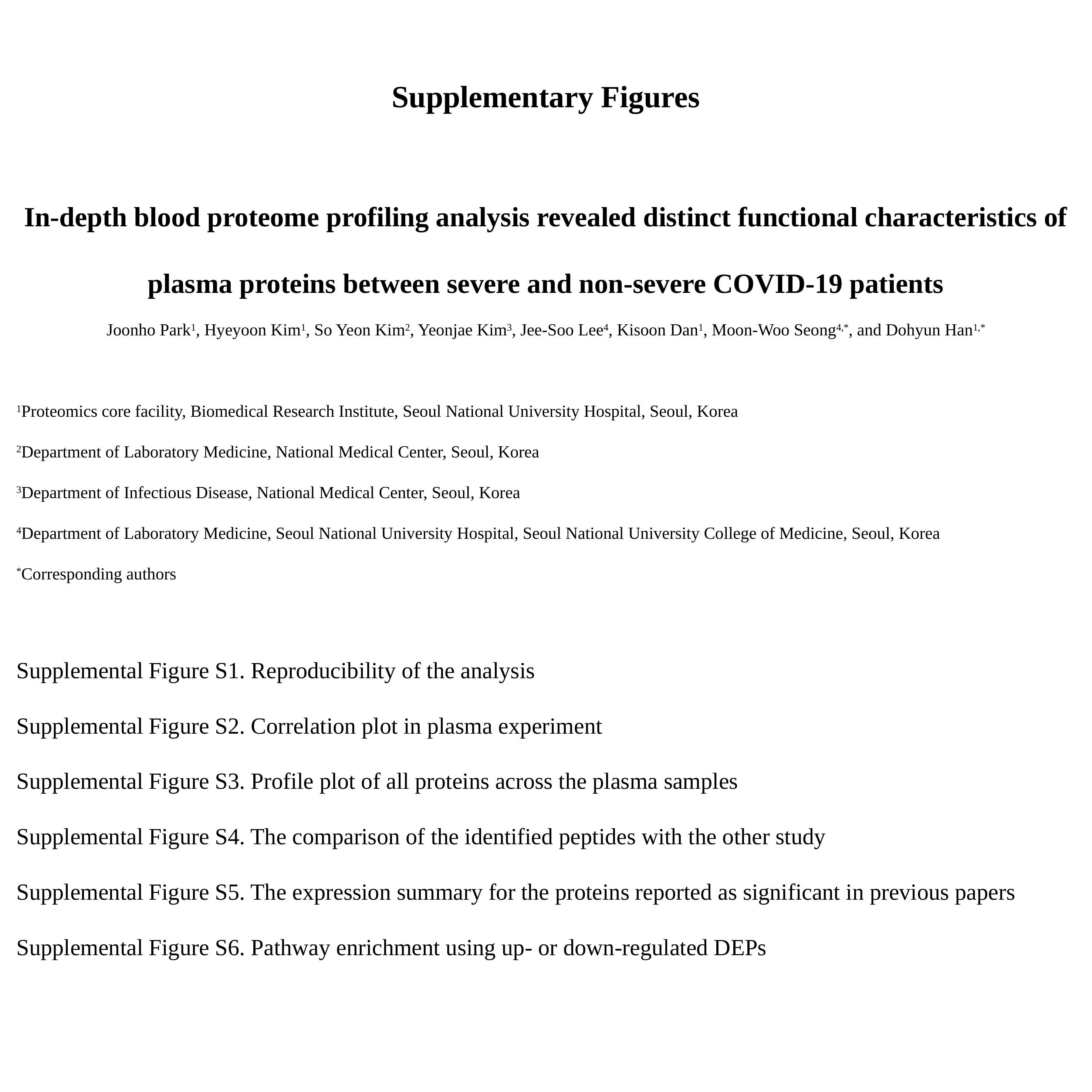

Supplementary Figures
In-depth blood proteome profiling analysis revealed distinct functional characteristics of plasma proteins between severe and non-severe COVID-19 patients
Joonho Park1, Hyeyoon Kim1, So Yeon Kim2, Yeonjae Kim3, Jee-Soo Lee4, Kisoon Dan1, Moon-Woo Seong4,*, and Dohyun Han1,*
1Proteomics core facility, Biomedical Research Institute, Seoul National University Hospital, Seoul, Korea
2Department of Laboratory Medicine, National Medical Center, Seoul, Korea
3Department of Infectious Disease, National Medical Center, Seoul, Korea
4Department of Laboratory Medicine, Seoul National University Hospital, Seoul National University College of Medicine, Seoul, Korea
*Corresponding authors
Supplemental Figure S1. Reproducibility of the analysis
Supplemental Figure S2. Correlation plot in plasma experiment
Supplemental Figure S3. Profile plot of all proteins across the plasma samples
Supplemental Figure S4. The comparison of the identified peptides with the other study
Supplemental Figure S5. The expression summary for the proteins reported as significant in previous papers
Supplemental Figure S6. Pathway enrichment using up- or down-regulated DEPs

## Slide 2
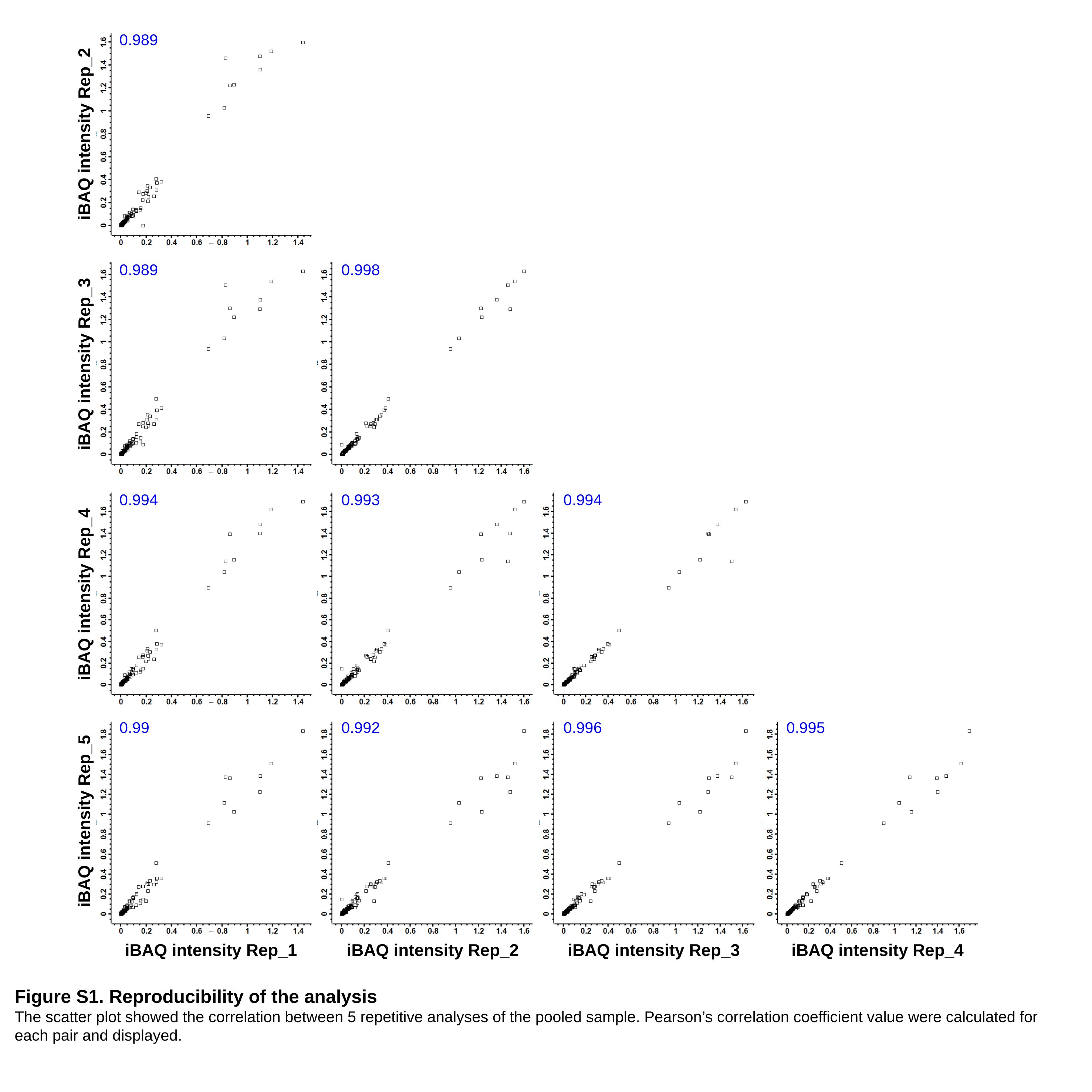

0.989
iBAQ intensity Rep_2
0.989
0.998
iBAQ intensity Rep_3
0.994
0.993
0.994
iBAQ intensity Rep_4
0.99
0.992
0.996
0.995
iBAQ intensity Rep_5
iBAQ intensity Rep_1
iBAQ intensity Rep_2
iBAQ intensity Rep_3
iBAQ intensity Rep_4
Figure S1. Reproducibility of the analysis
The scatter plot showed the correlation between 5 repetitive analyses of the pooled sample. Pearson’s correlation coefficient value were calculated for each pair and displayed.

## Slide 3
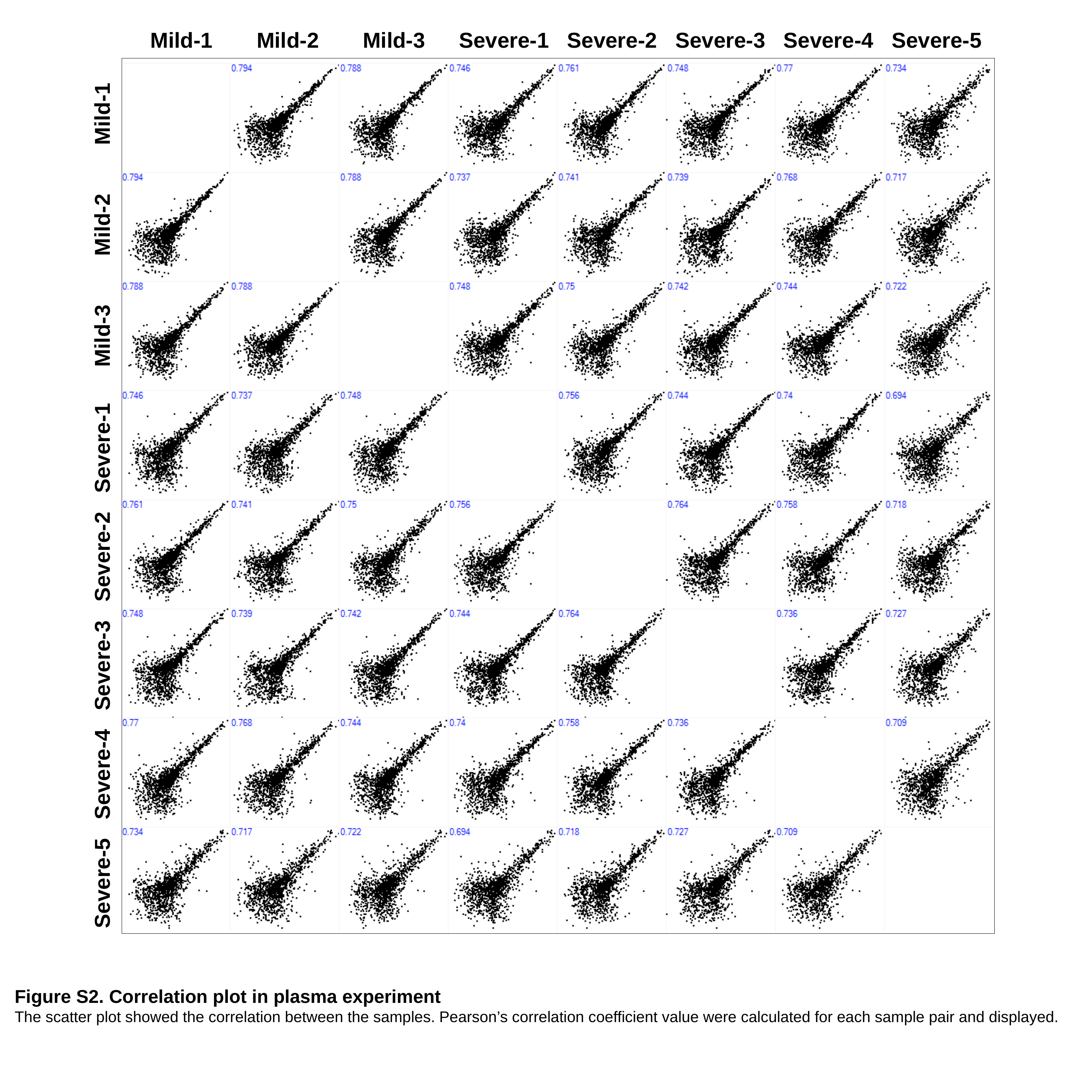

Mild-1
Mild-2
Mild-3
Severe-1
Severe-2
Severe-3
Severe-4
Severe-5
Mild-1
Mild-2
Mild-3
Severe-1
Severe-2
Severe-3
Severe-4
Severe-5
Figure S2. Correlation plot in plasma experiment
The scatter plot showed the correlation between the samples. Pearson’s correlation coefficient value were calculated for each sample pair and displayed.

## Slide 4
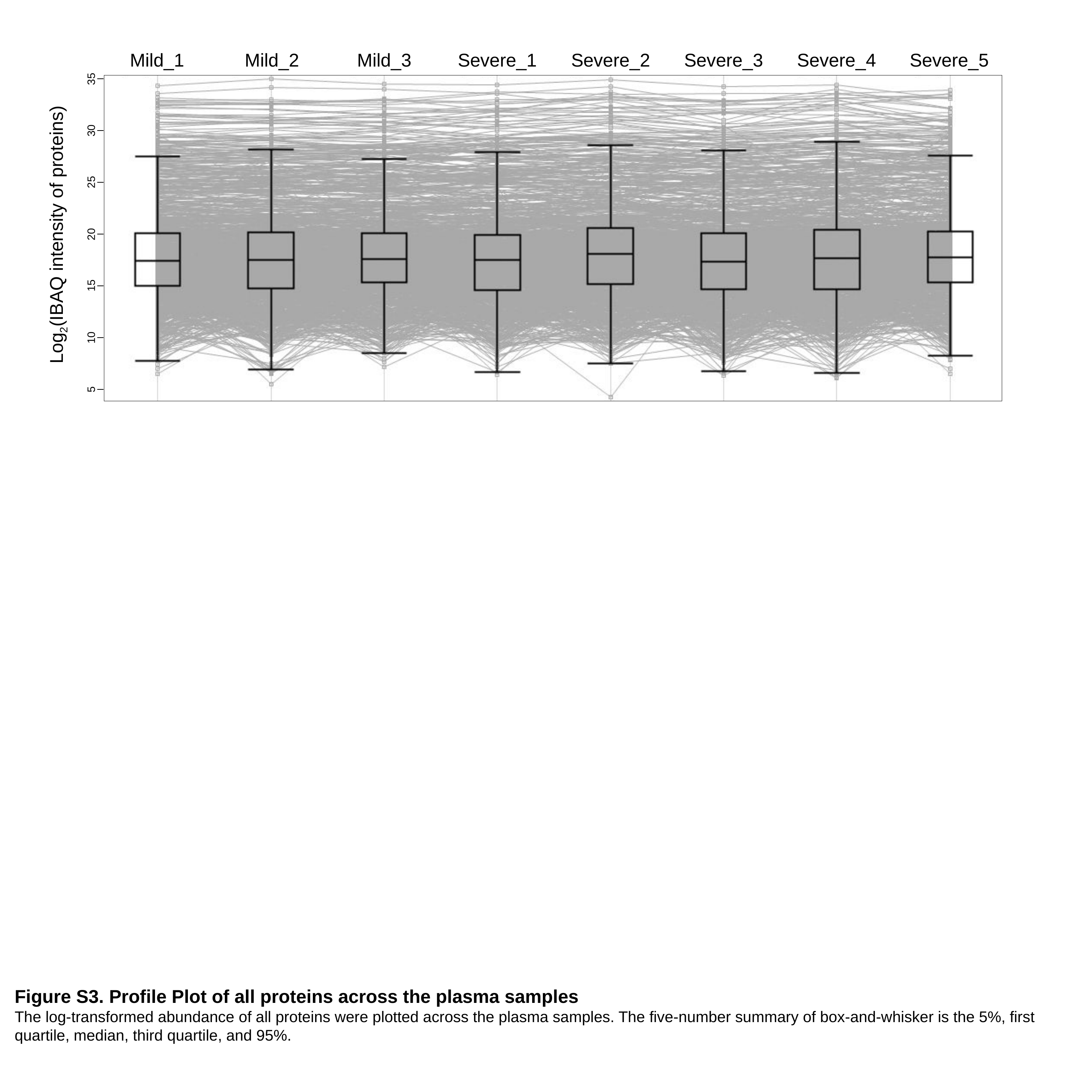

Mild_1
Mild_2
Mild_3
Severe_1
Severe_2
Severe_3
Severe_4
Severe_5
35
30
25
Log2(IBAQ intensity of proteins)
20
15
10
5
Figure S3. Profile Plot of all proteins across the plasma samples
The log-transformed abundance of all proteins were plotted across the plasma samples. The five-number summary of box-and-whisker is the 5%, first quartile, median, third quartile, and 95%.

## Slide 5
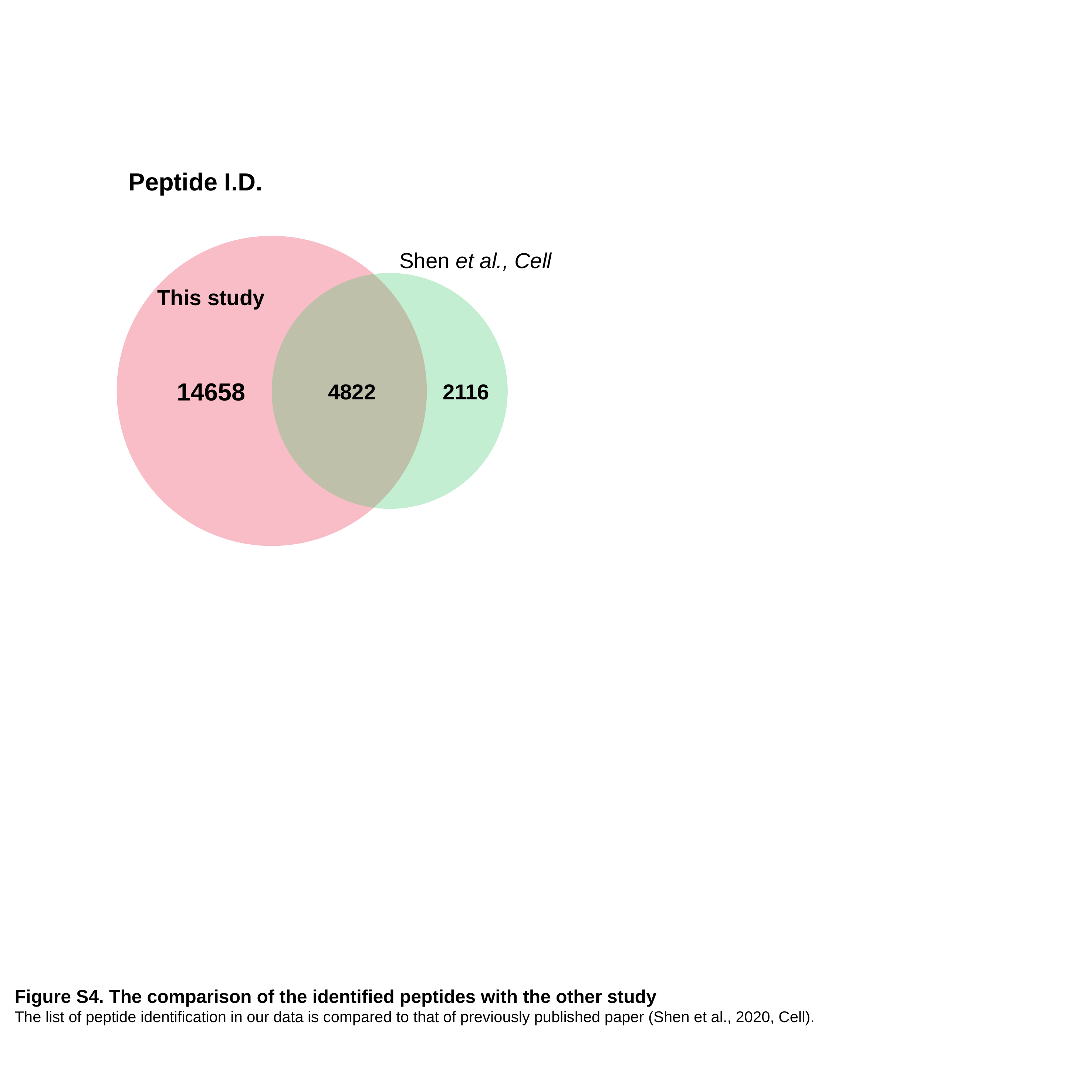

Peptide I.D.
Shen et al., Cell
This study
14658
4822
2116
Figure S4. The comparison of the identified peptides with the other study
The list of peptide identification in our data is compared to that of previously published paper (Shen et al., 2020, Cell).

## Slide 6
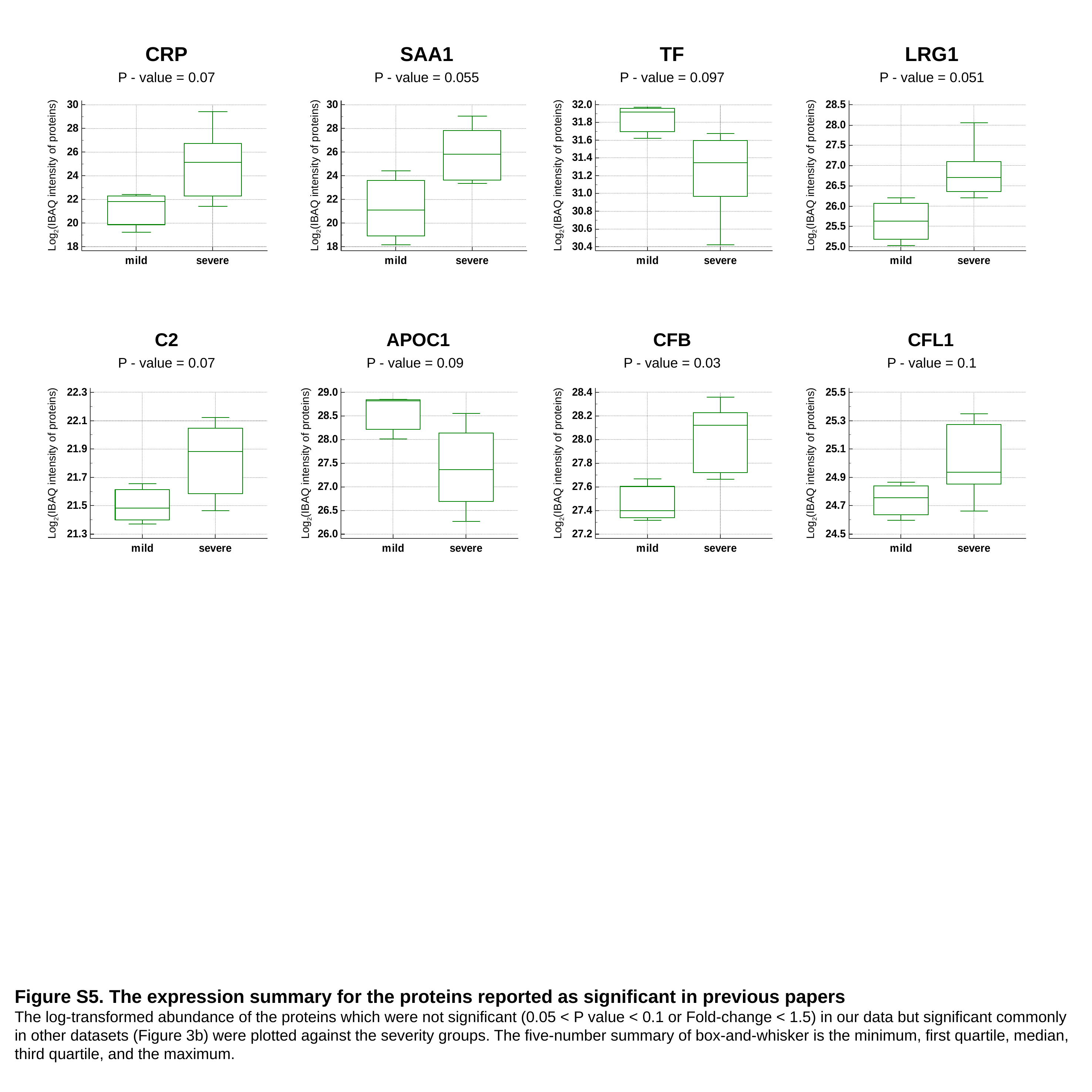

CRP
SAA1
TF
LRG1
P - value = 0.07
P - value = 0.055
P - value = 0.097
P - value = 0.051
Log2(IBAQ intensity of proteins)
Log2(IBAQ intensity of proteins)
Log2(IBAQ intensity of proteins)
Log2(IBAQ intensity of proteins)
C2
APOC1
CFB
CFL1
P - value = 0.07
P - value = 0.09
P - value = 0.03
P - value = 0.1
Log2(IBAQ intensity of proteins)
Log2(IBAQ intensity of proteins)
Log2(IBAQ intensity of proteins)
Log2(IBAQ intensity of proteins)
Figure S5. The expression summary for the proteins reported as significant in previous papers
The log-transformed abundance of the proteins which were not significant (0.05 < P value < 0.1 or Fold-change < 1.5) in our data but significant commonly in other datasets (Figure 3b) were plotted against the severity groups. The five-number summary of box-and-whisker is the minimum, first quartile, median, third quartile, and the maximum.

## Slide 7
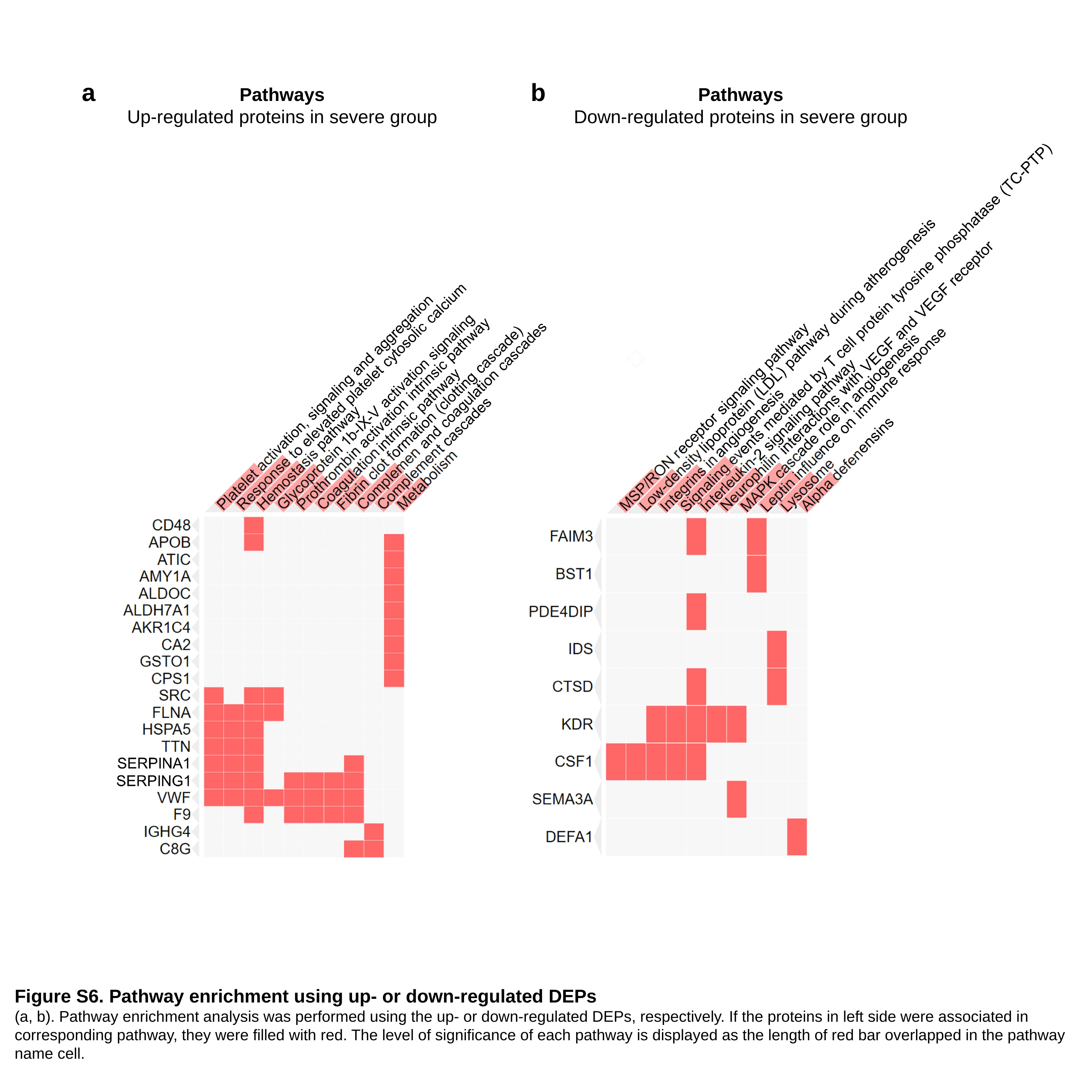

a
b
Pathways
Up-regulated proteins in severe group
Pathways
Down-regulated proteins in severe group
Figure S6. Pathway enrichment using up- or down-regulated DEPs
(a, b). Pathway enrichment analysis was performed using the up- or down-regulated DEPs, respectively. If the proteins in left side were associated in corresponding pathway, they were filled with red. The level of significance of each pathway is displayed as the length of red bar overlapped in the pathway name cell.
